# Supplementary material for: Little fast, little slow, should I stay or should I go? Adapting cognitive control to local-global temporal prediction across typical development
Source: PLoS One. 2023 Feb 24;18(2):e0281417. doi: 10.1371/journal.pone.0281417 (PMC9955637; doi:10.1371/journal.pone.0281417)
Supplement: S1 Table — We report the unstandardized regression coefficients (in logit scale), standard errors (SE), 95% confidence intervals (CI), degrees of freedom (df), and the associated statistic (t-test). (DOCX) [file pone.0281417.s001.docx]

**S1 Table.** **Results of LMM on log-transformed IES.**

|  | **log-IES** | | | | | |
| --- | --- | --- | --- | --- | --- | --- |
| *Predictors* | *Estimates* | *SE* | *CI* | *Statistic* | *p* | *df* |
| (Intercept) | 5.95 | 0.01 | 5.93 – 5.98 | 500.97 | **<0.001** | 41138.00 |
| adolescents | -0.13 | 0.02 | -0.16 – -0.09 | -7.73 | **<0.001** | 41138.00 |
| older children | -0.07 | 0.02 | -0.12 – -0.03 | -3.06 | **0.002** | 41138.00 |
| younger children | 0.04 | 0.02 | 0.00 – 0.08 | 2.04 | **0.042** | 41138.00 |
| fast-1 | -0.04 | 0.00 | -0.04 – -0.04 | -16.47 | **<0.001** | 41138.00 |
| slow-2 | -0.03 | 0.00 | -0.03 – -0.02 | -11.32 | **<0.001** | 41138.00 |
| fast-2 | 0.04 | 0.00 | 0.03 – 0.04 | 14.91 | **<0.001** | 41138.00 |
| SOA 1000 | 0.06 | 0.00 | 0.06 – 0.06 | 42.21 | **<0.001** | 41138.00 |
| adolescents * fast-1 | 0.04 | 0.00 | 0.03 – 0.05 | 12.28 | **<0.001** | 41138.00 |
| older children * fast-1 | 0.02 | 0.00 | 0.01 – 0.03 | 4.71 | **<0.001** | 41138.00 |
| younger children * fast-1 | -0.03 | 0.00 | -0.04 – -0.03 | -8.51 | **<0.001** | 41138.00 |
| adolescents * slow-2 | 0.01 | 0.00 | 0.00 – 0.02 | 3.15 | **0.002** | 41138.00 |
| older children * slow-2 | -0.00 | 0.00 | -0.01 – 0.01 | -0.62 | 0.535 | 41138.00 |
| younger children * slow-2 | -0.01 | 0.00 | -0.02 – -0.00 | -2.02 | **0.044** | 41138.00 |
| adolescents * fast-2 | -0.03 | 0.00 | -0.03 – -0.02 | -7.60 | **<0.001** | 41138.00 |
| older children * fast-2 | -0.00 | 0.00 | -0.01 – 0.01 | -0.51 | 0.608 | 41138.00 |
| younger children * fast-2 | 0.02 | 0.00 | 0.01 – 0.02 | 4.21 | **<0.001** | 41138.00 |
| adolescents * SOA 1000 | -0.00 | 0.00 | -0.01 – -0.00 | -2.55 | **0.011** | 41138.00 |
| older children * SOA 1000 | 0.02 | 0.00 | 0.01 – 0.02 | 6.23 | **<0.001** | 41138.00 |
| younger children * SOA 1000 | 0.00 | 0.00 | -0.00 – 0.01 | 1.11 | 0.268 | 41138.00 |
| fast-1 * SOA 1000 | 0.00 | 0.00 | -0.00 – 0.01 | 1.30 | 0.194 | 41138.00 |
| slow-2 * SOA 1000 | -0.00 | 0.00 | -0.01 – 0.00 | -1.88 | 0.060 | 41138.00 |
| fast-2 * SOA 1000 | 0.01 | 0.00 | 0.00 – 0.01 | 3.53 | **<0.001** | 41138.00 |
| adolescents * fast-1 * SOA 1000 | 0.02 | 0.00 | 0.01 – 0.03 | 6.33 | **<0.001** | 41138.00 |
| older children * fast-1 * SOA 1000 | -0.00 | 0.00 | -0.01 – 0.00 | -0.96 | 0.339 | 41138.00 |
| younger children * fast-1 * SOA 1000 | -0.01 | 0.00 | -0.02 – -0.00 | -2.75 | **0.006** | 41138.00 |
| adolescents * slow-2 * SOA 1000 | -0.01 | 0.00 | -0.01 – -0.00 | -2.01 | **0.045** | 41138.00 |
| older children * slow-2 * SOA 1000 | -0.01 | 0.00 | -0.02 – 0.00 | -1.71 | 0.088 | 41138.00 |
| younger children * slow-2 * SOA 1000 | 0.01 | 0.00 | 0.01 – 0.02 | 3.30 | **0.001** | 41138.00 |
| adolescents * fast-2 * SOA 1000 | -0.01 | 0.00 | -0.01 – 0.00 | -1.59 | 0.111 | 41138.00 |
| older children * fast-2 * SOA 1000 | 0.00 | 0.00 | -0.01 – 0.01 | 0.42 | 0.674 | 41138.00 |
| younger children * fast-2 * SOA 1000 | -0.00 | 0.00 | -0.01 – 0.00 | -1.22 | 0.221 | 41138.00 |
| σ^2^ | 0.06 | | | | | |
| τ_00_ | 0.03 _id_ | | | | | |
| ICC | 0.35 | | | | | |
| N | 266 _id_ | | | | | |
| Observations | 41172 | | | | | |
| Marginal R^2^ / Conditional R^2^ | 0.167 / 0.460 | | | | | |

We report the unstandardized regression coefficients (in logit scale), standard errors (*SE*), 95% confidence intervals (*CI*), degrees of freedom (*df*), and the associated statistic (*t*-test).
