# Supplementary material for: Little fast, little slow, should I stay or should I go? Adapting cognitive control to local-global temporal prediction across typical development
Source: PLoS One. 2023 Feb 24;18(2):e0281417. doi: 10.1371/journal.pone.0281417 (PMC9955637; doi:10.1371/journal.pone.0281417)
Supplement: S2 Table — For each contrast, we report the estimate (in logit scale), standard errors (SE), degrees of freedom (df), and the associated statistic (t-test). (DOCX) [file pone.0281417.s002.docx]

**S2 Table. Post-hoc contrasts of the *age group* main effect of the IES model.**

| **contrast** | **estimate** | ***SE*** | ***df*** | ***t*** | ***p*** |
| --- | --- | --- | --- | --- | --- |
| adults vs. adolescents | -0.053 | 0.034 | 263 | -1.55 | .407 |
| adults vs. older children | -0.166 | 0.027 | 263 | -6.19 | **< .001** |
| adults vs. younger children | -0.289 | 0.030 | 263 | -9.66 | **< .001** |
| adolescents vs. older children | -0.114 | 0.037 | 263 | -3.08 | **.012** |
| adolescents vs. younger children | -0.236 | 0.039 | 263 | -6.03 | **< .001** |
| older children vs. younger children | -0.123 | 0.033 | 263 | -3.70 | **.001** |

For each contrast, we report the estimate (in logit scale), standard errors (*SE*), degrees of freedom (*df*), and the associated statistic (*t*-test).
