# Supplementary material for: Little fast, little slow, should I stay or should I go? Adapting cognitive control to local-global temporal prediction across typical development
Source: PLoS One. 2023 Feb 24;18(2):e0281417. doi: 10.1371/journal.pone.0281417 (PMC9955637; doi:10.1371/journal.pone.0281417)
Supplement: S3 Table — For each contrast, we report the estimate (in logit scale), standard errors (SE), degrees of freedom (df), and the associated statistic (t-test). (DOCX) [file pone.0281417.s003.docx]

**S3 Table.** **Post-hoc contrasts of the *block sequence* main effect of the IES model.**

| **contrast** | **estimate** | ***SE*** | ***df*** | ***t*** | ***p*** |
| --- | --- | --- | --- | --- | --- |
| slow-1 vs. fast-1 | -0.012 | 0.004 | 40878 | -3.13 | **.009** |
| fast-1 vs. slow-2 | -0.064 | 0.004 | 40878 | -16.05 | **< .001** |
| slow-2 vs. fast-2 | 0.005 | 0.004 | 40879 | 1.35 | .529 |

For each contrast, we report the estimate (in logit scale), standard errors (*SE*), degrees of freedom (*df*), and the associated statistic (*t*-test).
