# Supplementary material for: Little fast, little slow, should I stay or should I go? Adapting cognitive control to local-global temporal prediction across typical development
Source: PLoS One. 2023 Feb 24;18(2):e0281417. doi: 10.1371/journal.pone.0281417 (PMC9955637; doi:10.1371/journal.pone.0281417)
Supplement: S4 Table — Only contrasts relative to long SOA intervals are reported. For each contrast, we report the estimate (in logit scale), standard errors (SE), degrees of freedom (df), and the associated statistic (t-test). (DOCX) [file pone.0281417.s004.docx]

**S4 Table.** **Post-hoc contrasts of the *age group* × *block sequence* × *SOA* interaction effect of the IES model.**

| **SOA** | **age group** | **contrast** | **estimate** | ***SE*** | ***df*** | ***t*** | ***p*** |
| --- | --- | --- | --- | --- | --- | --- | --- |
| 1000 | adults | slow-1 vs. fast-1 | -0.017 | 0.008 | 40878 | -2.315 | .095 |
|  |  | fast-1 vs. slow-2 | -0.013 | 0.008 | 40879 | -1.737 | .304 |
|  |  | slow-2 vs. fast-2 | -0.014 | 0.008 | 40879 | -1.779 | .283 |
|  | adolescents | slow-1 vs. fast-1 | 0.002 | 0.014 | 40878 | 0.174 | .998 |
|  |  | fast-1 vs. slow-2 | -0.041 | 0.014 | 40878 | -2.887 | **.020** |
|  |  | slow-2 vs. fast-2 | 0.014 | 0.014 | 40878 | 0.969 | .767 |
|  | older children | slow-1 vs. fast-1 | -0.022 | 0.010 | 40878 | -2.150 | .137 |
|  |  | fast-1 vs. slow-2 | -0.093 | 0.010 | 40878 | -9.177 | **< .001** |
|  |  | slow-2 vs. fast-2 | -0.011 | 0.010 | 40878 | -1.089 | .697 |
|  | younger children | slow-1 vs. fast-1 | -0.044 | 0.012 | 40878 | -3.632 | **.002** |
|  |  | fast-1 vs. slow-2 | -0.056 | 0.012 | 40878 | -4.619 | **< .001** |
|  |  | slow-2 vs. fast-2 | -0.031 | 0.012 | 40878 | -2.529 | .056 |

Only contrasts relative to long SOA intervals are reported. For each contrast, we report the estimate (in logit scale), standard errors (*SE*), degrees of freedom (*df*), and the associated statistic (*t*-test).
