# Supplementary material for: Little fast, little slow, should I stay or should I go? Adapting cognitive control to local-global temporal prediction across typical development
Source: PLoS One. 2023 Feb 24;18(2):e0281417. doi: 10.1371/journal.pone.0281417 (PMC9955637; doi:10.1371/journal.pone.0281417)
Supplement: S5 Table — For each model, we reported the unstandardized regression coefficients, standard errors (SE), 95% confidence intervals (CI), degrees of freedom (df), and the associated statistic (t-test). (DOCX) [file pone.0281417.s005.docx]

**S5 Table. Results of LMs on early and late delta scores.**

|  | **early delta scores** | | | | | | **late delta scores** | | | | | |
| --- | --- | --- | --- | --- | --- | --- | --- | --- | --- | --- | --- | --- |
| *Predictors* | *Estimates* | *SE* | *CI* | *Statistic* | *p* | *df* | *Estimates* | *SE* | *CI* | *Statistic* | *p* | *df* |
| (Intercept) | 6.12 | 0.28 | 5.57 – 6.67 | 21.75 | **<0.001** | 41164.00 | -0.31 | 0.27 | -0.84 – 0.22 | -1.16 | 0.247 | 41164.00 |
| adolescents | -15.34 | 0.39 | -16.11 – -14.58 | -39.37 | **<0.001** | 41164.00 | -1.99 | 0.38 | -2.72 – -1.25 | -5.30 | **<0.001** | 41164.00 |
| older children | -10.31 | 0.57 | -11.43 – -9.19 | -18.02 | **<0.001** | 41164.00 | -4.32 | 0.55 | -5.40 – -3.24 | -7.84 | **<0.001** | 41164.00 |
| younger children | 10.80 | 0.46 | 9.91 – 11.70 | 23.64 | **<0.001** | 41164.00 | 0.86 | 0.44 | -0.01 – 1.72 | 1.94 | 0.052 | 41164.00 |
| SOA 1000 | -1.89 | 0.28 | -2.44 – -1.34 | -6.72 | **<0.001** | 41164.00 | -6.39 | 0.27 | -6.92 – -5.85 | -23.57 | **<0.001** | 41164.00 |
| adolescents * SOA 1000 | -11.85 | 0.39 | -12.62 – -11.09 | -30.41 | **<0.001** | 41164.00 | -1.47 | 0.38 | -2.21 – -0.74 | -3.93 | **<0.001** | 41164.00 |
| older children * SOA 1000 | -2.84 | 0.57 | -3.96 – -1.72 | -4.96 | **<0.001** | 41164.00 | 3.41 | 0.55 | 2.33 – 4.49 | 6.20 | **<0.001** | 41164.00 |
| younger children * SOA 1000 | 11.11 | 0.46 | 10.21 – 12.01 | 24.30 | **<0.001** | 41164.00 | 3.71 | 0.44 | 2.85 – 4.58 | 8.44 | **<0.001** | 41164.00 |
| Observations | 41172 | | | | | | 41172 | | | | | |
| R^2^ / R^2^ adjusted | 0.097 / 0.097 | | | | | | 0.026 / 0.026 | | | | | |

For each model, we reported the unstandardized regression coefficients, standard errors (*SE*), 95% confidence intervals (*CI*), degrees of freedom (*df*), and the associated statistic (*t*-test).
