# Supplementary material for: Little fast, little slow, should I stay or should I go? Adapting cognitive control to local-global temporal prediction across typical development
Source: PLoS One. 2023 Feb 24;18(2):e0281417. doi: 10.1371/journal.pone.0281417 (PMC9955637; doi:10.1371/journal.pone.0281417)
Supplement: S6 Table — For each contrast, we report the estimate (in ms), standard errors (SE), degrees of freedom (df), and the associated statistic (t-test). (DOCX) [file pone.0281417.s006.docx]

**S6 Table. Post-hoc contrasts of the *age group* main effect of the early and late delta scores models.**

| **index** | **contrast** | **estimate** | ***SE*** | ***df*** | ***t*** | ***p*** |
| --- | --- | --- | --- | --- | --- | --- |
| early delta | adults vs. adolescents | -5.04 | 0.801 | 41164 | -6.29 | **< .001** |
|  | adults vs. older children | -26.15 | 0.636 | 41164 | -41.10 | **< .001** |
|  | adults vs. younger children | -30.19 | 0.716 | 41164 | -42.18 | **< .001** |
|  | adolescents vs. older children | -21.11 | 0.869 | 41164 | -24.29 | **< .001** |
|  | adolescents vs. younger children | -25.16 | 0.929 | 41164 | -27.08 | **< .001** |
|  | older children vs. younger children | -4.04 | 0.791 | 41164 | -5.11 | **< .001** |
| late delta | adults vs. adolescents | 2.33 | 0.771 | 41164 | 3.02 | **.013** |
|  | adults vs. older children | -2.85 | 0.612 | 41164 | -4.64 | **< .001** |
|  | adults vs. younger children | -7.44 | 0.689 | 41164 | -10.80 | **< .001** |
|  | adolescents vs. older children | -5.17 | 0.837 | 41164 | -6.18 | **< .001** |
|  | adolescents vs. younger children | -9.77 | 0.894 | 41164 | -10.92 | **< .001** |
|  | older children vs. younger children | -4.59 | 0.762 | 41164 | -6.03 | **< .001** |

For each contrast, we report the estimate (in ms), standard errors (*SE*), degrees of freedom (*df*), and the associated statistic (*t*-test).
