# Supplementary material for: Little fast, little slow, should I stay or should I go? Adapting cognitive control to local-global temporal prediction across typical development
Source: PLoS One. 2023 Feb 24;18(2):e0281417. doi: 10.1371/journal.pone.0281417 (PMC9955637; doi:10.1371/journal.pone.0281417)
Supplement: S7 Table — Only contrasts relative to long SOA intervals are reported. For each contrast, we report the estimate (in ms), standard errors (SE), degrees of freedom (df), and the associated statistic (t-test). (DOCX) [file pone.0281417.s007.docx]

**S7 Table. Post-hoc contrasts of the *age group* × *SOA* interaction effect of the early and late delta scores models.**

| **index** | **SOA** | **contrast** | **estimate** | ***SE*** | ***df*** | ***t*** | ***p*** |
| --- | --- | --- | --- | --- | --- | --- | --- |
| early delta | 1000 | adults vs. adolescents | 3.98 | 1.132 | 41164 | 3.51 | **.002** |
|  |  | adults vs. older children | -3.19 | 0.898 | 41164 | -3.55 | **.002** |
|  |  | adults vs. younger children | -14.76 | 1.009 | 41164 | -14.63 | **< .001** |
|  |  | adolescents vs. older children | -7.16 | 1.229 | 41164 | -5.83 | **< .001** |
|  |  | adolescents vs. younger children | -18.73 | 1.312 | 41164 | -14.29 | **< .001** |
|  |  | older children vs. younger children | -11.57 | 1.116 | 41164 | -10.37 | **< .001** |
| late delta | 1000 | adults vs. adolescents | 7.21 | 1.090 | 41164 | 6.619 | **< .001** |
|  |  | adults vs. older children | 2.34 | 0.864 | 41164 | 2.709 | **.034** |
|  |  | adults vs. younger children | -11.62 | 0.971 | 41164 | -11.970 | **< .001** |
|  |  | adolescents vs. older children | -4.87 | 1.182 | 41164 | -4.120 | **< .001** |
|  |  | adolescents vs. younger children | -18.83 | 1.262 | 41164 | -14.920 | **< .001** |
|  |  | older children vs. younger children | -13.96 | 1.074 | 41164 | -13.002 | **< .001** |

Only contrasts relative to long SOA intervals are reported. For each contrast, we report the estimate (in ms), standard errors (*SE*), degrees of freedom (*df*), and the associated statistic (*t*-test).
